# Multifocal neuropathy and Horner’s syndrome due to disseminated pyomyositis in an intravenous drug user

**DOI:** 10.1007/s10072-023-07024-z

**Published:** 2023-08-31

**Authors:** Aaron de Souza, Brian Z. Liew

**Affiliations:** 1 Department of Medicine, Launceston General Hospital, 274-280 Charles Street, Launceston, TAS 7250 Australia; 2 Faculty of Medicine, Launceston Clinical School, University of Tasmania, Launceston, TAS 7250 Australia

**Keywords:** Pyomyositis, Intravenous drug user, Neuropathy, Sciatic, Radial, Horner’s syndrome

## Abstract

**Background:**

The formation of abscesses with necrosis within large, striated muscles leads to pyomyositis, a condition relatively rarely encountered outside the tropics. Intravenous drug users and other immunocompromised individuals are predisposed toward this infection, which may occur due to local or haematogenous spread of infection to skeletal muscles previously damaged by trauma, exercise, or rhabdomyolysis.

**Methods:**

We report a young male intravenous drug user with rhabdomyolysis due to use of a synthetic opioid, in whom disseminated pyomyositis was detected following evaluation for sciatic and radial neuropathies and Horner’s syndrome and review available reports of peripheral nerve dysfunction in the setting of this uncommon infection. We searched online databases to identify all published reports on adult patients with pyomyositis complicated by peripheral nerve dysfunction.

**Conclusions:**

Peripheral nerve dysfunction may rarely occur via local spread of infection or compression from abscesses.

## Introduction

Pyomyositis, or the formation of abscesses with necrosis within large, striated muscles is a rare condition in temperate climates. It is clinically heterogeneous, and the broad range of often vague clinical features coupled with the rarity of the condition commonly lead to a delay in diagnosis and appropriate treatment. Risk factors include immune compromise (human immunodeficiency virus infection, diabetes mellitus), trauma, or intravenous drug use [1–4]. Peripheral nerve dysfunction may rarely occur via local spread of infection or compression from abscesses, and neuropathic weakness should be distinguished from that due to muscle damage. We present a young male intravenous drug user (IVDU) who developed radial and sciatic nerve palsies and Horner’s syndrome due to disseminated pyomyositis in the setting of drug-induced rhabdomyolysis and review available reports of peripheral nerve dysfunction in the setting of this uncommon infection.

## Case report

A 25-year-old university student with a premorbid history of bipolar disorder, anxiety, and recreational substance use was admitted to the Emergency Department after an accidental overdosage of the synthetic opioid protonitazene, being found surrounded by drug injecting paraphernalia in his room, drowsy, and unable to move. Evaluation revealed deranged liver and renal functions, a high-anion gap mixed metabolic and respiratory acidosis, hyperkalaemia, left lower and right middle lobe pneumonia, and elevated creatine kinase (88,401 IU/L, reference range 30–120). Indurated swellings were noted over the arms, with multiple needle tracks, cellulitis of the left leg and thrombophlebitis over all limbs. Following intubation and mechanical ventilation for hypoxia and agitation, he was commenced on piperacillin-tazobactam, vancomycin, fluid therapy, and diuresis with furosemide. Renal function improved without dialysis, and pneumonia responded to antibiotics. No focal neurological deficits were evident at this time. Marked swelling in the left arm and cubital fossa prompted ultrasound examination, which demonstrated subcutaneous oedema, basilic vein thrombosis, and increased echogenicity in the brachialis muscle with central low attenuation suggesting focal myositis. There was no evidence of compartment syndrome or endocarditis. Attempts at percutaneous drainage were unsuccessful, and flucloxacillin was added.

**Fig. 1:**
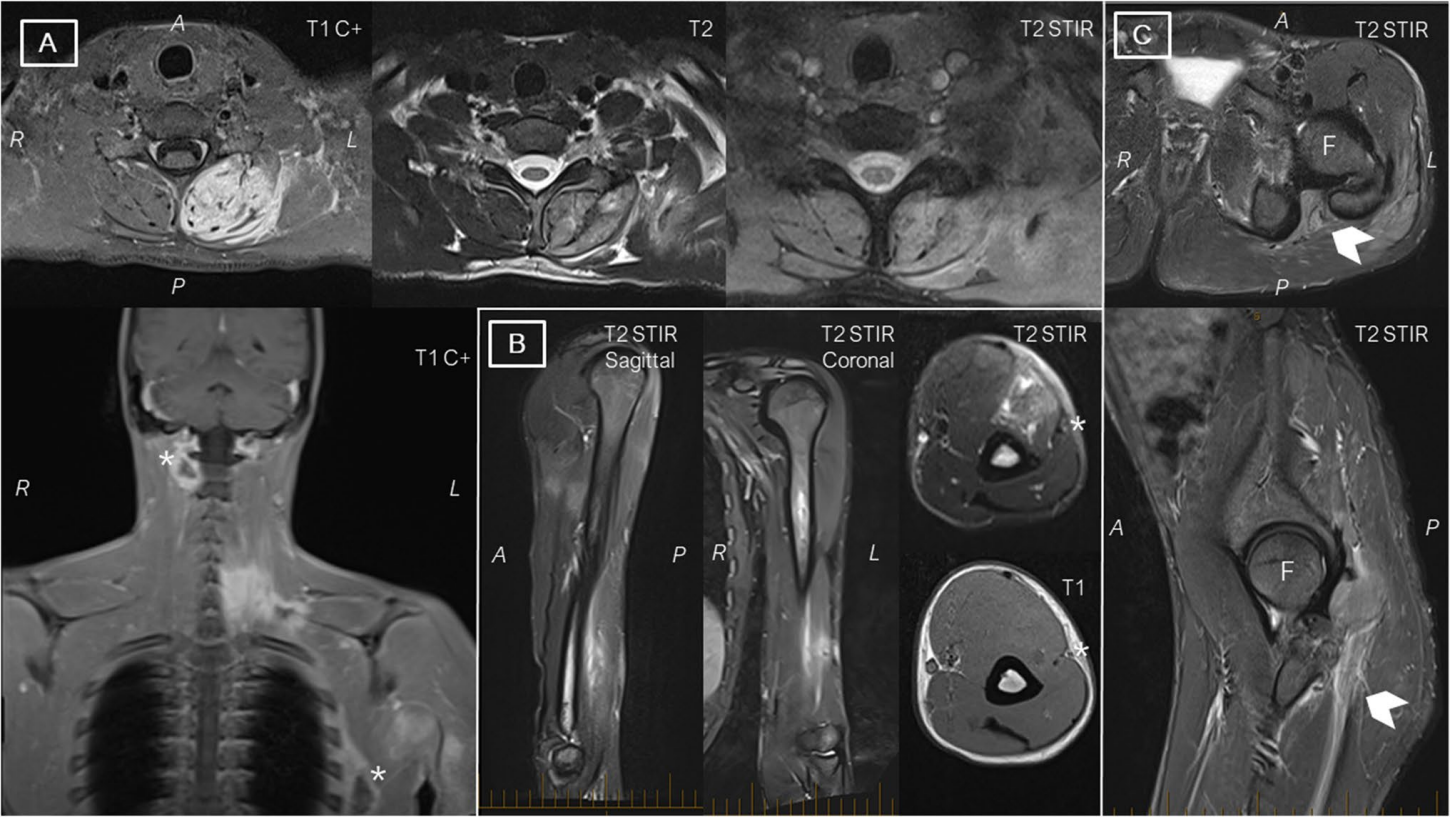
**A** T2-weighted, T2-STIR, and T1-weighted postcontrast (T1C +) axial images at the level of C7 vertebra show muscle swelling, contrast enhancement, and relatively mild T2-signal change in the left paraspinal muscles. The lack of diffusion restriction and of prominent T2-weighted signal change indicates that this is likely a phlegmon rather than a true abscess. Note additional abscesses identified in the left chest wall and the right paraspinal regions (asterisks) on the T1C + coronal image. **B** T2 STIR images showing signal change in the brachialis, in close proximity to the neurovascular bundle (asterisk) corresponding to the electrophysiological localization of the patient’s radial neuropathy. **C** Axial and sagittal T2-STIR images through the left hip demonstrating signal change in the obturator internus and gluteus medius with a T2-STIR hyperintense collection at the musculotendinous junction in the gluteus maximus. Minor compression of the sciatic nerve with extensive signal change consistent with inflammatory neuritis is seen (chevrons). A: anterior; F: femoral head; L: left; R: right; P: posterior

CK declined rapidly to 10,043 in 48 h, and to 2971 over a further 72 h. After extubation and weaning of sedation, he complained of pain, paraesthesia, and weakness of the left arm and a left foot drop, with left eyelid droop. Neurological evaluation confirmed left Horner’s syndrome with ptosis, miosis, and anhidrosis over the left face; left radial palsy with weakness of forearm radial-innervated muscles and sensory loss over the distribution of the superficial radial nerve; and severe left knee flexion and foot weakness with anesthesia over the distributions of the left superficial and deep peroneal, sural, and tibial nerves. MRI performed on day 23 of admission showed normal appearances of the brain and spine with multifocal areas of myositis and hyperintense signal in the left sciatic nerve (Fig. 1). Nerve conduction studies done 29 days after admission demonstrated a severe left sciatic neuropathy with absent tibial and peroneal motor and sural and superficial peroneal sensory responses, along with a radial neuropathy localizing to the distal left arm (reduced radial motor and sensory amplitudes, slowing of motor nerve conduction velocity between the spiral groove and elbow). EMG was not performed due to the risk of dissemination of infection [5, 6]. No obvious collection amenable to surgical drainage was noted. Multiple attempts at aspiration of abscess contents under ultrasound or CT guidance were unsuccessful, and blood cultures performed daily for three weeks did not indicate a likely pathogen. Empirical antibiotics were continued, with good recovery of left upper limb weakness and Horner’s syndrome, and partial improvement in the foot weakness. Repeat imaging of the left sciatic nerve after five months demonstrated normal nerve signal with no impingement. Signal change consistent with denervation was noted in the semimembranosus, biceps femoris, tibialis anterior, gastrocnemius, and soleus.

## Discussion

Pyomyositis is rare outside the tropics, occurring mainly in immunosuppressed persons or intravenous drug users (IVDUs). Impaired humoral and cellular immunity, abnormal skin microflora, and the repeated introduction of nonsterile fluids via contaminated needles all predispose IVDUs to pyomyositis [4, 7–9]. Direct muscle trauma or vigorous exercise has been implicated in 22 to 67% of pyomyositis cases [10, 11] and alterations in muscle structure play a role in seeding of infection from transient bacteraemia, creating an abnormal microenvironment that facilitates growth of pathogenic bacteria [1, 9, 12, 13]. Abscesses may develop via haematogenous spread or from nearby bone or soft tissue. The most common sites of abscess formation are the quadriceps, gluteus, gastrocnemius, iliopsoas, and shoulder and arm muscles [1, 3–5, 9, 12, 13]. Disseminated involvement is not uncommon, being described in up to 40%, particularly in the presence of immune compromise [1, 5, 10–12, 14]. Bacterial infection leads to skeletal muscle oedema, and often subsequent phlegmon (enhancing infected granulation tissue) formation and then an abscess, characterized by a focal fluid collection containing inflammatory cells, bacteria, and necrotic tissue debris surrounded by vascular, inflamed connective tissue [15, 16]. Pyomyositis may evolve through an initial “invasive” stage, with swollen, indurated and woody muscles to a suppurative or purulent stage with fluctuant muscle swellings, to a “late” stage associated with severe toxicity and possible local extension to bone or joints [1, 4, 9, 12, 17].

MRI is invaluable in the diagnosis of pyomyositis, demonstrating relevant pathology in all patients, even in the early stages [7, 12, 15, 18–21]. Infected muscles appear enlarged and oedematous with heterogeneously increased T2-weighted (T2W) signal, and intermediate T1-weighted (T1W) signal [7, 9, 12]. Fluid collections, if present, are hyperintense on T2W images and hypointense on T1W imaging with diffusion restriction [7, 9, 12, 15, 22, 23]. The rim of the abscess demonstrates T1W hyperintense and T2W hypointense signal with peripheral contrast enhancement [7, 19, 20]. Subcutaneous oedema and unorganized phlegmonous collections may be seen in soft-tissue adjacent to areas of active muscle inflammation [7]. The presence of peripherally-enhancing abscesses may indicate the need for surgical drainage in addition to antibiotic therapy, and imaging may additionally demonstrate enhancement of deep fascia and septic arthritis or osteomyelitis secondary to local extension [9, 12, 15].

Our patient with disseminated pyomyositis had likely drug-induced rhabdomyolysis, and we postulate that haematogenous dissemination occurred from a soft tissue infection to the lungs and to previously compromised muscles. Given prompt antibiotic treatment, it is perhaps unsurprising that serial blood cultures were negative. Blood cultures may be positive in as few as 5–10% of cases in the tropics, rising to 20–30% in temperate regions [2, 11]. Attempts at aspiration from the muscle abscesses detected on imaging were unsuccessful—this may be due to the early stage of the infection or the presence of unorganized phlegmon rather than a true abscess. In a pediatric series [12], organized abscesses were present in less than half of patients. Muscle aspiration is negative in the initial stage of the infection but may yield results in the purulent stage [9]. The responsible bacterium was identified only in around 50% of patients in various series [12, 24]. *Staphylococcus aureus* accounts for 95% of such cases in the tropics and 70% in temperate areas. Other organisms include streptococcal species, anaerobes, and Gram-negative bacilli, the latter being more common among immunosuppressed patients [25, 26]. Treatment is guided by the results of blood cultures or abscess aspiration, if available, and monitoring of clinical response and inflammatory markers in blood gauges the response or lack thereof. While the duration of antibiotic treatment varies, intravenous antibiotics followed by oral therapy are usually given for a total of 3–8 weeks, whether the abscess is drained or not [13, 25].

## Peripheral nerve dysfunction due to pyomyositis

Local extension or neural compression may result in peripheral nerve palsies, which often improve with antibiotic treatment [27]. We searched PubMed, EMBASE, Google, and Google Scholar databases using the keywords “pyomyositis” and “neuropathy” (or “nerve palsy”) to identify all published reports on adult patients with pyomyositis complicated by peripheral nerve dysfunction. The last search was performed on March 01, 2023.

Table 1 lists previously reported cases of peripheral nerve dysfunction associated with pyomyositis in adult and adolescent patients. The sciatic nerve may be involved with pyomyositis of the pelvic muscles, including the pyriformis and glutei and more rarely the iliacus or obturator internus. The peroneal nerve and the lateral pterygoid branch of the mandibular ramus of the trigeminal nerve have also been affected by abscesses in contiguous muscles. Our patient is unusual in that his disseminated muscle infection produced multifocal neuropathies. While preganglionic Horner’s syndrome may occur due to pneumonia, mediastinitis, or retropharyngeal abscess, to our knowledge, this is the first report of Horner’s syndrome due to pyomyositis. The presence of ptosis, miosis, and anhidrosis implicate the phlegmon in the region of the cervical sympathetic ganglia as demonstrated on imaging as the cause of the patient’s Horner’s syndrome, involving the second-order neurons of the sympathetic pathway [28]. The temporal course of the neuromuscular deficits, with onset after improvement in his rhabdomyolysis, as well as the lack of clinical evidence of compartment syndrome implicate compression due to pyomyositis as the likely cause of his nerve palsies. While EMG would have undoubtedly aided the accurate localization of nerve injury, the risk of dissemination of infection was deemed unacceptably high [5, 6].

**Table 1:** Reports of peripheral nerve dysfunction due to pyomyositis in adult and adolescent patients

Reference	Year	Age(years)/sex	Nerve involved	Muscle(s) involved	Postulated mechanism	Organism	Treatment
Present case	2023	25/M	Sciatic, radial, sympathetic chain	Disseminated	Intravenous drug use, rhabdomyolysis, cellulitis	Not identified	Empirical antibiotics
[29]	2021	41/M	Sciatic	Piriformis, glutei	Diabetes mellitus	*S aureus* from pus	Surgical drainage, antibiotics
[30]	2019	37/M	Sciatic	Piriformis	Not described	*S aureus* from aspirate	CT-guided aspiration, antibiotics
[31]	2016	49/M	Sciatic	Obturator internus	Subacute bacterial endocarditis, chronic liver disease, recent skin graft	*Staphylococcus haemolyticus* from blood culture	Antibiotics
[32]	2013	51/M	Mandibular division of trigeminal	Lateral pterygoid	Odontogenic infection following dental extraction	Not identified	Surgical drainage, antibiotics
[33]	2013	42/F	Sciatic	Piriformis	Not described	Not identified	Surgical drainage, antibiotics
[34]	2012	31/F	Sciatic	Piriformis	Ventouse delivery with epidural analgesia and episiotomy, vaginal wall haematoma	Group B Streptococci from blood and vaginal swab; *E. coli* and mixed anaerobes from vaginal swab	Antibiotics
[35]	2012	18/M	Sciatic	Piriformis, iliopsoas, sacro-iliac joint	Playing rugby	*S aureus* from blood	Antibiotics
[36]	2009	45/M	Sciatic	Iliopsoas, piriformis, glutei	Gluteal injury while skiing	*S aureus* from blood and pus	Antibiotics, CT-guided drainage
[26]	2008	45/F	Sciatic	Piriformis, iliacus, pelvic abscess	Not described	Not identified	Empirical antibiotics
[37]	2008	26/M	Sciatic	Gluteus medius and minimus	Intense exercise, infected mosquito bite	*S aureus* from pus	Surgical drainage, antibiotics
[38]	2007	18/F	Sciatic	Piriformis	Endometritis following unsafe abortion	*S aureus* from blood culture and CT-guided aspiration	Antibiotics following aspiration
[39]	2004	30/F	Sciatic	Piriformis, gluteus medius	Dilatation and curettage for missed abortion	*S aureus* from curettage samples	Empirical antibiotics
[40]	2003	40/F	Sciatic	Gluteus medius, adductors	Long lie after drug overdose, rhabdomyolysis, infected pressure ulcers	*Enterobacter aerogenes* and coagulase-positive Staphylococci from pus	Surgical drainage, antibiotics
[27]	2003	16/F	Peroneal	Tibialis anterior	Not described	Not identified	Antibiotics
[41]	1998	16/F	Sciatic, sacral plexus	Piriformis	Not described	*S aureus* from blood and on aspiration of pus	Antibiotics following aspiration
[42]	1998	17/M	Sciatic	Piriformis	Vigorous swimming in a hot pool	*P mirabilis* on blood cultures	Antibiotics
[43]	1995	22/F	Sciatic	Piriformis, iliacus, SIJ	Forceps delivery with epidural analgesia, perineal laceration	*E faecalis* and Group B Streptococci from vaginal swab	Antibiotics
[44]	1992	42/M	Sciatic	Piriformis	Respiratory tract infection	*S aureus* from pus	Surgical drainage, antibiotics

*M*, male; *F*, female

Among previously reported cases, similar to our patient, pyomyositis occurred in the setting of relatively minor muscle injury, via haematogenous seeding or spread from local infection. *S. aureus*, Group B Streptococci, *Enterococcus faecalis*, *Escherichia coli*, coagulase-positive Staphylococci, anaerobes, *Enterobacter aerogenes*, and *Proteus mirabilis* have been identified, but no organism was isolated in 22% of patients. Surgical drainage or aspiration of pus was required in about half, but other patients improved solely with antibiotic therapy. Prompt identification of an infectious process causing the nerve dysfunction is therefore very important, and this must be differentiated from more common conditions including intervertebral disc prolapse or pressure palsies that might produce similar clinical findings.

## Data Availability

Data sharing not applicable—no new data generated.
